# Lifestyles and Quality of Life of People with Mental Illness During the COVID-19 Pandemic

**DOI:** 10.1007/s10597-023-01095-0

**Published:** 2023-02-13

**Authors:** Giada Tripoli, Sofia Lo Duca, Laura Ferraro, Uzma Zahid, Raffaella Mineo, Fabio Seminerio, Alessandra Bruno, Vanessa Di Giorgio, Giuseppe Maniaci, Giovanna Marrazzo, Crocettarachele Sartorio, Alessandra Scaglione, Daniele La Barbera, Caterina La Cascia

**Affiliations:** 1 Department of Biomedicine, Neuroscience, and Advanced Diagnostics, University of Palermo, Via G. La Loggia 1, 90129 Palermo, Italy; 2 Department of Health Promotion, Mother and Child Care, Internal Medicine and Medical Specialties, University of Palermo, Piazza delle Cliniche 2, 90127 Palermo, Italy; 3 Department of Psychosis Studies, Institute of Psychiatry, Psychology, and Neuroscience, King’s College London, De Crespigny Park, Denmark Hill, SE5 8AF London, UK; 4 Unit of Psychiatry, University Hospital “Paolo Giaccone”, Via G. La Loggia 1, 90129 Palermo, Italy; 5 Department of Psychiatry, University of Oxford, Warneford Hospital, Warneford Ln, Headington, OX3 7JX Oxford, UK

**Keywords:** Quality of life, Lifestyles, Mental illness, COVID-19

## Abstract

The COVID-19 pandemic has had a significant impact on the quality of life (QoL), daily lifestyle, and mental health of people suffering from a mental disorder. This study aimed to investigate the effects of the prolongation of the COVID-19 emergency on QoL and lifestyles in a sample of 100 outpatients at the Psychiatry Unit in Palermo University Hospital, Italy. QoL was measured through the 12-item Short Form Survey and the COV19-Impact on Quality of Life. Lifestyle changes during the pandemic were measured through the lifestyle change questionnaire. The majority of participants reported a great impact of COVID-19 on the QoL, and almost half reported worsened lifestyles. Worsened lifestyles were predictive of both poor mental and physical health related QoL. These results suggest that people with mental illness need interventions targeting lifestyles, and the mental health service in Italy should adjust to the ongoing pandemic, developing virtual treatments.

## Introduction

Since the outbreak of the Sars-Cov-2 virus in January 2020, the COVID-19 pandemic has forced governments worldwide to take measures to contain the spread of the virus, which in turn drastically changed people’s lives and everyday habits. On 9th March 2020, Italy was the first European country to announce a national lockdown, with the prohibition of all non-essential activities and confinement at home (Decree of the President of the Council of Ministers 9 March 2020). Following this, the Italian government imposed a range of containment measures, such as physical distancing, use of personal protective equipment (PPE), remote learning and home-based work, no travel outside the region of residence, suspension of bars, restaurants, and retail activities (Decree of the President of the Council of Ministers no. 64 of 11 march 2020–Decree of the President of the Council of Ministers n.265 of 24 October 2020). These measures varied across the national territory according to the epidemic risk, which was based on indicators of the effective reproduction number (Rt), and hospital and intensive care unit (ICU) admission rates (Decree of the President of the Council of Ministers 3 November 2020).

Empirical evidence suggests that the COVID-19 pandemic and the subsequent control measures have had a significant impact on the quality of life (QoL), daily lifestyle, and mental health of the general population. Studies have found higher levels of depressive symptoms and distress compared to before the outbreak (Wang et al., 2020; Resnick et al., 2020; Fountoulakis et al., 2021). Individuals suffering from a psychiatric disorder were more at risk of having psychological and health-related consequences because of COVID-19, since they showed a greater stress vulnerability than the general population and have less strategies to cope with unexpected life events, such as the outbreak of a pandemic (Holmes et al., 2020; Pfefferbaum & North, 2020; Sukut & Ayhan Balik, 2021).

Furthermore, mental health services in Europe underwent severe disruptions during the first wave of the pandemic (World Health Organization, 2020). In Italy, on 10th April 2020, less than two months after the Italian patient zero, 98,273 people tested positive for the virus and 31,739 were hospitalized with symptoms, of which 3497 were in intensive care (Ministry of Health, 2020). To face the increasing demands of hospital beds for patients infected with COVID-19, entire wards in general hospitals, including psychiatric ones, were reconverted to COVID-19 units (De Girolamo et al., 2020). Outpatient units were forced to drastically limit appointments to the most urgent cases and offer only support via phone calls to the others, whereas day centers for rehabilitation programs were mostly closed. Patients in full residential programs were confined in the facilities with very limited or no possibility to leave (De Girolamo et al., 2020; D’Agostino et al., 2020; Burrai et al., 2020). The disruptions faced by mental health services along with home confinement and overall containment measures had an impact on the daily habits and quality of life of individuals with psychiatric disorders (Hao et al., 2020; Li et al., 2021; De Girolamo et al., 2020). Lifestyles have substantially changed among people with Severe Mental Illness (SMI) during the first wave: they exercised less, experienced weight gain, changed their sleep routine, and increased their tobacco use, raising their risk for metabolic syndrome and premature death (Chapman et al., 2021; Solé et al., 2021).

A cross-sectional study by Liu et al., (2020) surveyed a sample of 898 U.S. young adults during the first phase of the COVID-19 pandemic (March–May 2020). They found higher levels of depression, anxiety, post-traumatic stress disorder (PTSD) symptoms, sleep disturbances, and a poorer QoL overall in those with a pre-existing mental health diagnosis compared to those reporting no diagnosis. To investigate the impact of the COVID-19 pandemic on QoL, Repišti et al., (2020) validated a short new measure COV19-QoL in the Balkans on patients with severe mental illness (n = 201) and participants from the general population (n = 1346) during the first wave.

To date, Italy has experienced three waves of the COVID-19 pandemic, and most of the studies in the literature were carried out during the first wave. The present study aimed to investigate the effects of the prolongation of COVID-19 on QoL and lifestyles in an Italian sample of mental health service users. We aimed to (a) estimate QoL and the impact of the pandemic on QoL in the psychiatric population, comparing our results with those obtained by Repišti et al., (2020); (b) examine lifestyle habits and related modifications due to the pandemic; (c) examine whether lifestyle habits predict mental health-related quality of life.

## Methods

### Participants

This study was conducted between March 2021 and June 2021. One hundred participants were recruited from the outpatient service at the Psychiatry Unit of the University Hospital “Paolo Giaccone” (Palermo, Italy). Participants provided informed written consent and data were stored and treated anonymously. Ethical approval was obtained from Comitato Etico Palermo 1 (24/02/2021 n.2/2021).

Inclusion criteria were: (1) age 18–65; (2) main diagnosis of psychiatric disorder; (3) capability to understand the content of the informed consent and sign it; (4) at least one contact with the service in the previous three months. Exclusion criteria were: (1) psychiatric disorders due to organic condition; (2) moderate or severe intellectual disability.

All respondents were interviewed through a sociodemographic information form, which included questions on socio-demographic and economic factors, health, and housing conditions (number of cohabitants and house size – number of rooms). It also asked if respondents, family members, friends, or relatives had tested positive for COVID-19.

All patients were informed of the study aims and procedures and signed the consent in-person, eighty-four completed the assessment face-to-face (at the psychiatry unit clinics). Sixteen participants could not complete the assessment on the day, and agreed to complete over a telephone interview and through an online questionnaire (Google Forms).

## Quality of Life Assessment

### 12-item Short-Form Health Survey (SF-12)

SF-12 (Ware et al., 1996) is a self-evaluation test with 12 questions designed to assess a patient’s physical and mental wellbeing. It evaluates some health concepts including limitations in physical activities due to health problems, limitations in social activities due to physical or emotional problems, limitations in normal role activities due to health physical problems, pain, general mental health (psychological distress and well-being), limitations in normal role-playing activities due to emotional problems, vitality, and perceptions of general health. The score consists of a Physical Composite Score (SF-12 PCS) and Mental Composite Score (SF-12 MCS). Ruotolo and colleagues (2021), approximate reliability is estimated using Cronbach’s alpha coefficient for the elements most strongly associated with physical health and for those most strongly associated with mental health (item 1,8,9,10 considered as reverse value). In our study, for PCS *α* ***=*** 0.818; for MCS α = 0.789. The SF-12 appears to be a psychometrically sound instrument for measuring the health-related quality of life of people with SMI (Salyers et al., 2000).

### COV-19 – Impact on Quality of Life (COV19-QoL)

The COV19-QoL scale (Repišti et al., 2020) measures the impact of the current COVID-19 pandemic on people, in terms of perceived decrease in quality of life. Validation of the scale was conducted both on non-clinical and clinical samples, and the questionnaire was administered online though Google Forms in the original study. This scale has good reliability (Cronbach’s alpha coefficient of the scale for the data from the clinical sample was equal to 0.86) and continuous validity. The items evaluate how the person thinks about changes in their QoL due to COVID-19 using a 5-point Likert scale on the level of agreement, from 1 – “totally disagree” to 5 – “completely agree”.

In our study, the internal consistency of the scales is high (Cronbach’s α = 0.835). The Kaiser–Meyer–Olkin Sample Adequacy Test (KMO) is used to check validity. The Kaiser–Meyer–Olkin Sample Adequacy Test (KMO) is used to verify validity (as in the test validation study, Repišti et al., 2020), and the assumed value (0.826) is excellent.

### Adaptation of the Lifestyle Change Questionnaire

The questionnaire, originally developed by Cancello et al., (2020), included 31 items. The revised version contains 18 questions (multiple-choice, single choice, numerical) on reference data (age, sex, education, working conditions, presence of chronic diseases, smoking habits, hours of weekly physical activity, weight, and height) and changes in daily habits during the pandemic period (weight, sleep quality, physical activity, cigarette consumption, appetite, food purchase and intake, diet quality). Some example items: “How do you evaluate the quality of your nutrition compared to before isolation for COVID-19?“, or “Since the pandemic restrictions have been in place, how would you define your night sleep?”, or “In this pandemic period, compared to before lockdown, can you do physical activity?”.

The following variables were investigated:

- Sleep Quality (the presence of symptoms of insomnia, increased or decreased sleep duration, the perception of sleep was more or less restful).
- Food Habits and Nutrition (improved and worsened the quality of the diet; increased and decreased the food consumption, if it was planned the food supply, if it was prepared the list of foods to buy, which categories of foods was bought, needs underlying the main food choices, weight changes);
- Physical Activity (how often physical activity was performed);
- Smoking Habits (increased or decreased the cigarette consumption).

### Data Analysis

Data were analysed using IBM SPSS Version 26.0 for Windows. The validity and reliability of the quantitative tools were examined. Internal reliability was assessed using Cronbach’s reliability (α). Principal component analysis (PCA) explored the underlying structure of the final item set in the COV19-QoL and the Kaiser-Meyer-Olkin Sample Adequacy Test (KMO) was used to check validity.

Descriptive statistics (mean, frequency, standard deviations) of all study variables were computed. A one-sample *t*-test was performed to compare the mean of the total score and each item of COV19-QoL, with the values assumed by normative samples (clinical or not clinical) of the reference literature (Repišti et al., 2020).

A two-sample *t*-test was used to compare patients with SMI and patients with other psychiatric disorders on MCS (SF-12), PCS (SF-12) scores, and the COV19-QoL total score. Kruskal–Wallis test was used to compare people with SMI to the other patients on COV19-QoL items.

A two-sample *t*-test was used to assess pandemic related changes and compare groups based on dichotomous variables of quality of sleep, food habits and nutrition, and physical activity from the Adaptation of the Lifestyle Change Questionnaire on MCS (SF-12) and PCS (SF-12) scores. Linear regression models were used to investigate whether sleep quality, food quality, and adopting a sedentary lifestyle were significant predictors of MCS (SF-12) and PCS (SF-12) scores, controlling for age, sex, marital status, domestic situation, SMI or not SMI.

## Results

### Sample Characteristics

**Fig. 1:**
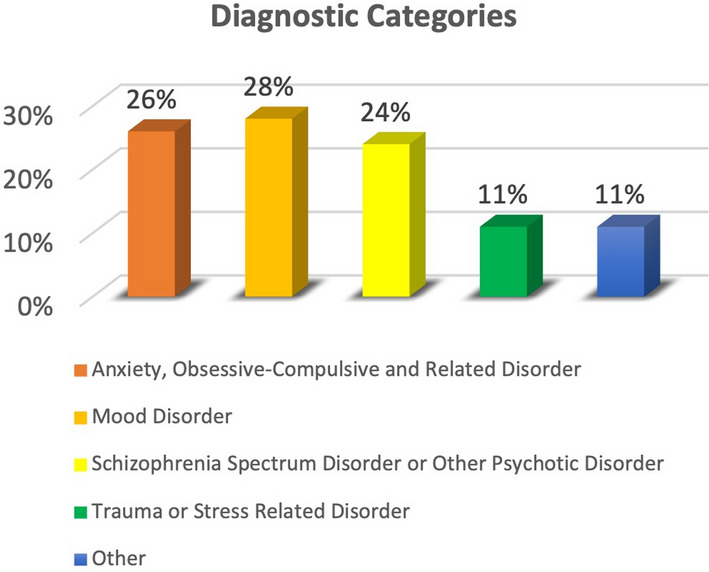
Diagnostic Categories distribution (N = 100)

One hundred patients were assessed. The socio-demographic characteristics are shown in Table 1. The main diagnoses were: Mood Disorders (28%), Anxiety, Obsessive-Compulsive and Related Disorder (26%) and, Schizophrenia Spectrum Disorder or Other Psychotic Disorder (24%) (Fig. 1).

**Table 1:** Socio-demographic characteristics of the sample (N = 100)

Variables	N%
Age mean (SD) = 43.1 (13.2)	
18–30	22
30–45	26
45–65	52
Gender	
Female	57
Male	43
Education level	
Low	41
Medium	47
High	12
Occupation	
Employed	21
Unemployed	70
Student	9
Marital status	
Single	41
Married/Living with someone	51
Separated/divorced/Widowed	8
Domestic situation	
Living alone	8
Living with partner/children/both	50
Living with parents /relatives / both	40
Other	2
Crowding index	**1.4**
BMI kg/m2	
Underweight	5
Normal weight	37
Overweight	26
Obese	32

**Fig. 2:**
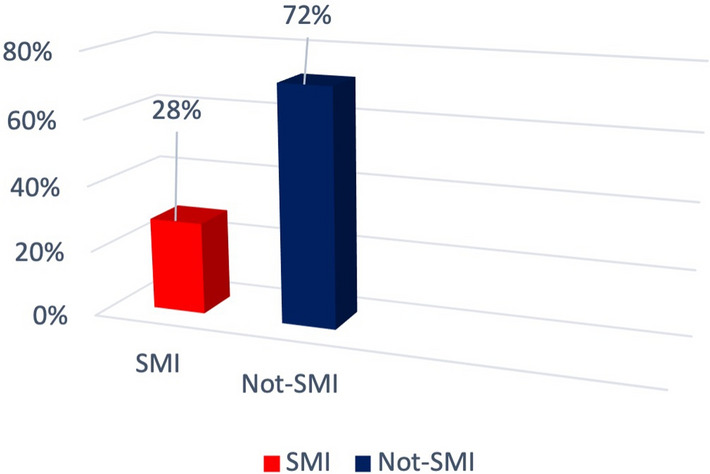
SMI vs. Not-SMI Diagnoses (N = 100)

Furthermore, participants were grouped by their diagnosis as per medical records into SMI or non-SMI categories (Fig. 2), according to Repišti et al. (2020), including a primary diagnosis of schizophrenia spectrum disorders (ICD-10 codes F20–F29), or bipolar disorder (ICD-10 codes F30, F31, F34.0). Seven patients had tested positive for COVID-19 and 59% of the entire sample had experienced a positive case among relatives and friends. Among those who belonged to the category of workers (N = 21) 57% continued to go to work, while 19% stopped working due to COVID-19 and another 19% started working from home.

## SF-12 - Short-Form-Health Survey

The values assumed by our sample are shown in Table 2. It is interesting to note that there is a difference between the average score assumed on the PCS-12 (M = 42.9) and the average score assumed on the MCS-12 (M = 34.5).

**Table 2:** SF-12 values

		PCS	MCS
Mean		42.9	34.5
Range		22.1–64.7	14.7–64.7
Percentiles	25	34.5	24.2
	50	44.1	32.2
	75	52.1	45.1
SD		10.9	11.9

## COV19-QoL

The questionnaire values assumed by our sample are shown in Table 3. Participants experienced a greater impact of the pandemic on the degree of perceived tension (item 4 “I feel more tense than before” M = 3.6) and a lower impact on the level of personal safety (item 6 “I feel at risk for my safety” M = 2.9), evaluated through the COV19-QoL (Repišti et al., 2020) on a 5-point Likert scale. We also tried to divide the values obtained in the reactive into percentiles. From the comparison between the clinical samples, a significant difference is found for all mean values (p < .0001) (Table 4), however, the differences with the non-clinical sample are significant only for the items relating to the quality of life and mental health (item 1, p < .007; item 2, p < .0001; item 3, p < .343; item 4, p < .0001; item 5, p < .0001; item 6, p < .100, total score, p < .04) (Table 5).

**Table 3:** COV-19-QoL values

Due to the spread of the coronavirus.	M	SD
… I think my quality of life is lower than before … I think my mental health has deteriorated … I think my physical health may deteriorate … I feel more tense than before … I feel more depressed than before … I feel that my personal safety is at risk	3.6 3.4 3.1 3.6 3.3 2.9	1.3 1.3 1.2 1.7 1.3 1.3
COV-19-QoL (total score)	3.3	0.3

**Table 4:** Comparison between the sample mean on the COV19QoL with the mean values assumed by clinical sample of Repišti et al., (2020)

*COV-19 QoL*	Mean sample	Mean clinical sample	P value
Item 1	3.6	3.1	p < .000
Item 2	3.4	2.1	p .<0.000
Item 3	3.1	2.5	p < .000
Item 4	3.6	2.6	p < .000
Item 5	3.3	2.1	p < .000
Item 6	2.9	2.1	p < .000

**Table 5:** Comparison between the sample mean on the COV19QoL with the mean values assumed by non-clinical sample of Repišti et al., (2020)

*COV-19 QoL*	Mean sample	Mean Non-clinical sample	P value
Item 1	3.6	3.3	p < .074
Item 2	3.4	2.6	p < .000
Item 3	3.1	3.02	p < .343
Item 4	3.6	3.1	p < .000
Item 5	3.3	2.7	p < .000
Item 6	2.9	2.7	p < .100

## SMI vs. non-SMI Categories and QoL

Suffering from a SMI was associated with higher MCS-12 score (SMI mean = 40.6, SD = 12.8 vs. Not-SMI mean = 32.1, SD = 10.6; t=-3.4; p = .0011) and PCS-12 score, although not significantly (SMI mean = 45, SD = 10.6 vs. Not-SMI mean = 42.1, SD = 10.9; t=−1.2; p = .2350). Furthermore, SMI status had less COV19-QoL total score than Not-SMI (SMI mean = 17.5, SD = 4.4 vs. Not-SMI mean = 20.9, SD = 5.6; t = 2.9; p = .0040). Regarding each COV19-QoL item, SMI diagnosis differed from not-SMI on item 1 (“I think my quality of life is lower than before”) (SMI median = 3, IQR = 2–4 vs. Not-SMI median = 4, IQR = 3–5; χ² = 7.4, p = .0067), item 2 (“I think my mental health has deteriorated”) (SMI median = 2, IQR = 2–4 vs. Not-SMI median = 4, IQR = 3–5; χ²=11.2, p = .0008) and item 5 (“I feel more depressed than before”) (SMI median = 2, IQR = 2–4 vs. Not-SMI median = 4, IQR = 3–5; χ² =6.8, p = .0089).

## Adaptation of the Lifestyle Change Questionnaire

**Fig. 3:**
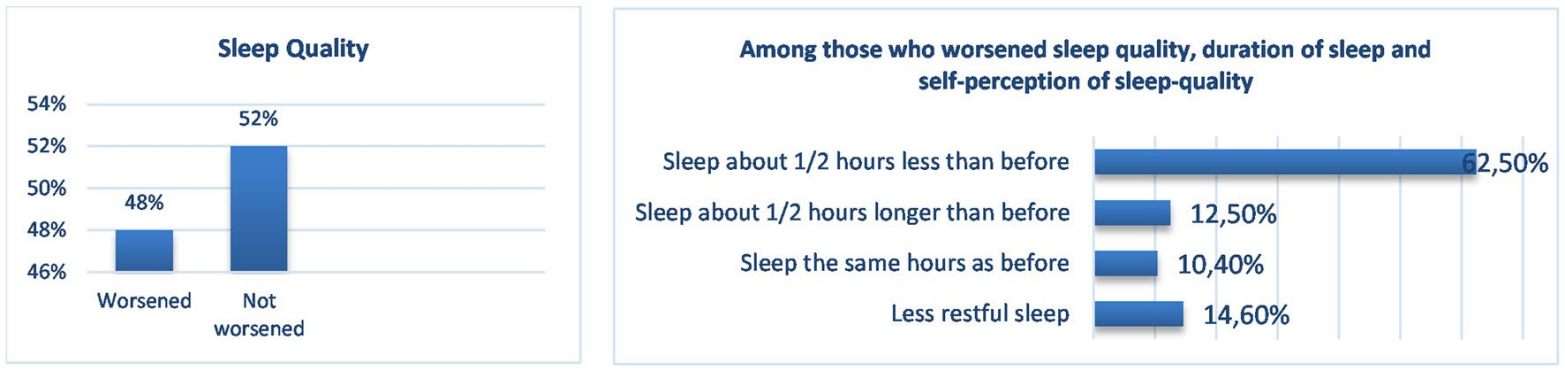
Sleep Quality

**Fig. 4:**
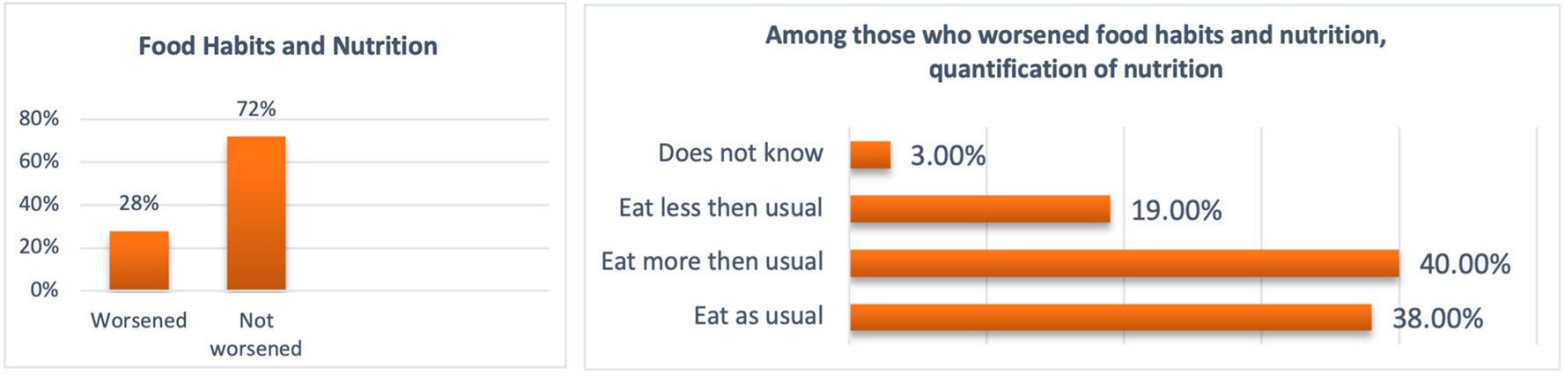
Nutrition Quality

**Fig. 5:**
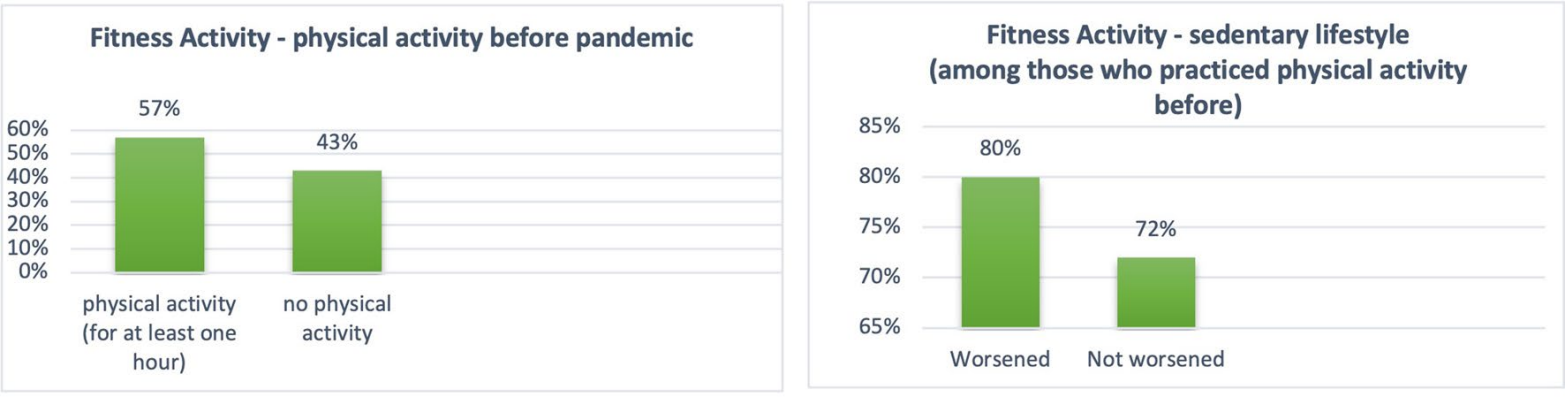
Fitness Activity

**Fig. 6:**
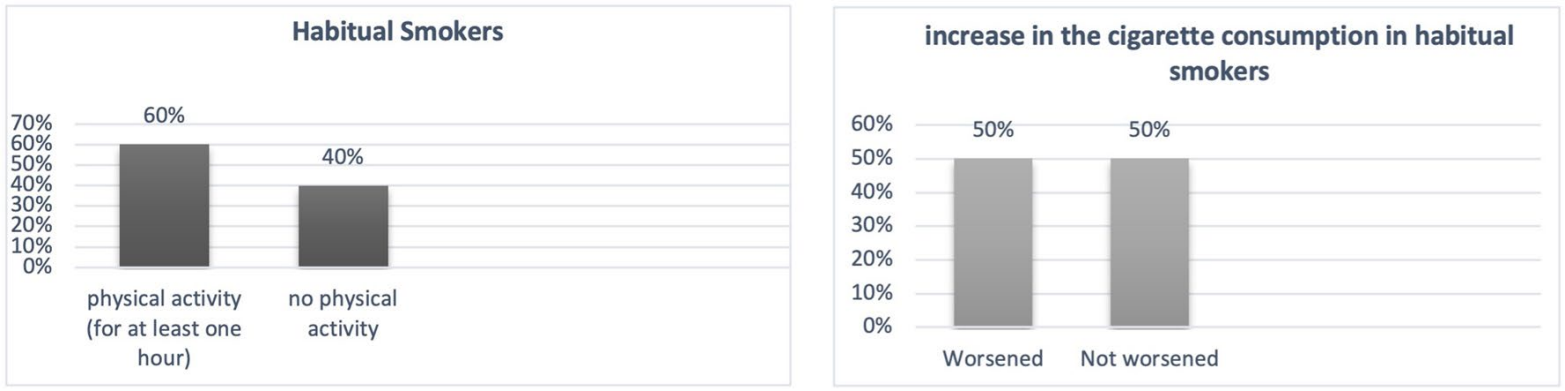
Smoking Habits

Due to the pandemic and related measures for containing the virus, 48% of the sample reported worse quality of sleep (Fig. 3), 28% considered their nutrition quality worsen (Fig. 4), and among those who were physically active before the pandemic (57% of the total), 76% reduced exercise or became inactive (Fig. 5). Among habitual smokers, only 50% increased their cigarette consumption (Fig. 6).

## Lifestyles Changes and SF-12

Changing lifestyles for worse was associated with a lower MCS-12 mean score compared to no changing or changing for better (sleep quality: 30.9, SD = 10.9 vs. 37.7, SD = 11.9, t=−2.9, p = .0037; diet quality: 30.2, SD = 9.7 vs. 36.1, SD = 12.3, t=−2.3, p = .0246; sedentary lifestyle: 31.9, SD = 11.5 vs. 42.4, SD = 9.3, t=−4.02, p = .0001). Mean scores of the PCS-12 were also lower when worsening their lifestyles relative to no change in lifestyle (sleep quality: 38.6, SD = 9.7 vs. 46.9, SD = 10.4, t=−4.1, p = .0001; diet quality: 39.4, SD = 9.3 vs. 44.3, SD = 11.2, t=−2.1, p = .0422; sedentary lifestyle: 41, SD = 11 vs. 48.9, SD = 7.9, t=−3.3, p = .0015).

Linear regression models confirmed a poor diet (B=−5.9, SE = 2.4, 95% CI −10.7 to −1.2, p = .015) and a sedentary lifestyle (B=−9.2, SE = 2.6, 95% CI −14.3 to −4.1, p = .001) as predictors of lower MCS-12, after adjusting for covariates. Linear regression also confirmed prediction of PCS-12 by worse sleep (B=−6.6, SE = 2, 95% CI −10.6 to −2.6, p = .001), diet (B=−5.6, SE = 2.2, 95% CI −9.9 to −1.3, p = .012), and sedentary behaviour (B=−5.6, SE = 2.4, 95% CI −10.4 to −0.8, p = .024).

## Discussion

This study set out to investigate the effects of the lengthening of the COVID-19 emergency and the related restrictions on the QoL and the lifestyles of mental health service users. Pre-pandemic psychiatric research reported that patients with SMI have a reduced QoL than the general population (Berghöfer et al., 2020). Among people with psychiatric disorders, those with SMI had deficits in health related QoL relative to those who suffered from other mental illnesses (Saarni et al., 2010). Studies that evaluated QoL among people diagnosed with mental health disorders, during the first wave of the pandemic, showed a poorer health related QoL and a negative impact of the pandemic on well-being (Liu et al., 2020, Repisti et al., 2020, Fiorenzato et al., 2021, Czysz et al., 2021). In line with these results, the majority of our study’s participants felt that their QoL worsened considerably due to the COVID-19 pandemic, especially due to the perceived level of tension. In addition, similar to Liu et al., (2020), our sample reported on average a worse QoL related to mental health than to physical health. However, those with SMI reported better mental health related QoL than those with other diagnoses. Furthermore, subjects with SMI perceived a lesser impact of the pandemic on their QoL compared to not-SMI patients. Two studies conducted in the first few months of the pandemic found a modest impact on perceived well-being (Fahy et al., 2021) or even an improvement in perceived well-being (Pinkham et al., 2020). Even before the COVID-19 spread, people with severe psychiatric pathology had poorer personal and social functioning (i.e., repetitive routines and few social relationships) (Velthorst et al., 2017). As reported by Fahy et al., (2021) it is possible that they experienced a less drastic change in their living habits during the early months of the pandemic than in the following stages (Table 4).

Similar to Repisti et al. (2020), we found a negative impact of the COVID-19 pandemic on QoL, however, our participants reported a worse QoL than the reference study for both clinical and non-clinical sample. This could be due to the extended state of emergency experienced in Italy, which could have impacted QoL more than during the first lockdown. A reason could be the drastic disruptions of mental health services for people with a diagnosis of mental disorder due to the pandemic and lockdown. The Italian health system has faced an unprecedented crisis, and mental health services have also been affected worldwide and in Italy (De Girolamo et al., 2020).

Our study sample was selected among outpatients from the Palermo University Hospital. In our hospital, the psychiatric ward was closed, from March 2020 until June 2021, to meet the high demand of admissions for patients with COVID-19. Outpatient activities (psychiatric appointments, psychotherapeutic and psychosocial interventions) were suspended during the first wave and subsequently interrupted, by the regional rules or the management’s hospital directives. Nevertheless, it was possible to keep some patients engaged through phone or video calls. However, not all patients were able to access appointments online, due to a lack of technical skills, as also reported by Spanakis et al., (2021). Studies show that interruption of treatments or a lack of continuity has negative consequences in terms of stress for people with psychiatric problems (De Girolamo et al., 2020). Whereas ensuring continuity of care leads to lower levels of stress (Burrai et al., 2020). Regarding their lifestyles, most of the sample reported worsened quality of sleep, almost a third considered their nutrition quality to be affected and among those who were physically active before the pandemic almost all reduced exercise or became inactive. Half of the sample had an increase in weight. Since the pandemic, food choices have changed for most of the sample and have been partly determined by the need to reduce costs. Only half of those who smoke habitually have increased the consumption of cigarettes. In this study a worsening of nutrition quality, and having a sedentary life were significantly associated with perceiving a worse mental health related QoL.

In pre-pandemic literature, the influence of lifestyles on QoL was investigated in people with schizophrenia. Findings suggested that sleep quality is associated with better QoL as well as physical activity. (Costa et al., 2018). A recent study by Solè et al. (2021), investigating the effects of COVID-19 pandemic effects, showed that people with severe mental illness reported weight gain and sleep disturbances during lockdown. A decrease in physical activity during the first wave of COVID-19 in people with bipolar disorder compared to healthy controls was also reported by Sperling et al., (2021).

Poor adherence to healthy lifestyle habits is not an unpredicted conclusion. There is considerable evidence that people with severe mental illness have lower levels of healthy lifestyle habits than the general population (Speyer et al., 2019; Naslund et al., 2017; Stubbs et al., 2018). Nonetheless, the lack of psychosocial interventions focused on increasing healthy behaviors during the lockdown could have worsened patients’ lifestyles choices, leading in turn to increase their risk for metabolic syndrome and cardiovascular mortality (Sohn et al., 2021).

## Limitations and Strengths

Our study has a number of limitations. We relied on self-report measurements, which are widely used and are common tools to collect data, however, it is possible that participants may recall information inaccurately. Nevertheless, the SF-12 is a tool that has good psychometric properties (Kodraliu et al., 2001) and the COV19-QoL has been considered a valid scale to evaluate the pandemic impact on QoL (Repisti et al., 2020). Another limitation is the heterogeneity of the sample, which included different diagnoses, however the current study aimed to investigate the effect of COVID-19 in mental health service users. We cannot make an inference regarding causality because of the cross-sectional design, therefore future longitudinal studies are needed .

This study has several strengths. There are a limited number of studies investigating the effects of COVID-19 on the QoL and lifestyles of psychiatric patients, therefore the present study contributes to the field (Chapman et al., 2021; Peckham et al., 2021; Solé et al., 2021; Sperling et al., 2021). Moreover, we provide evidence after the first wave of COVID-19 pandemic and lockdown, which is still a period little investigated.

## Conclusions and Implications

This study highlighted the effect of the COVID-19 on the lives of people with mental illness and the subsequent need for interventions on specific topics such as the identification of stress factors, the detection of early signs of recurrence, the learning of coping strategies and stress management techniques (e.g., through mindfulness-based programs) (Guan et al., 2021). Lifestyles interventions, especially targeting physical activity, diet and sleep hygiene, should be implemented to contrast inactivity and weight gain caused by restrictions, which required spending more time at home than usual (Solé et al., 2021). Moreover, the pandemic requires some adjustment to the usual methods of providing psychiatric care, psychological support, and rehabilitation (Guan et al., 2021).

Studies on the effectiveness of telepsychiatry—the delivery of psychiatric assessment and care using information and communication technologies (Shore, 2013)—show that, where accessibility to services is limited, it is possible to consider remote methods (like telephone and video calls) as an alternative to traditional interventions in an effort to ensuring adherence to psychopharmacological medication and psychosocial treatments (Chen et al., 2020; Guan et al., 2021).

In order to limit the effects of social isolation on QoL and psychological health of people with mental illness, online treatment should be provided not only individually but also in a group format (Guan et al., 2021).

Indeed some countries have offered telemedicine support to their populations (Liu et al., 2020; Benudis et al., 2022; Hunsinger et al., 2021). In Italy, only one study reported a successful clinical experience of virtual treatment for patients suffering from psychiatric disorders (Fagiolini et al., 2020). There are limited RCTs to date to informatively compare virtual interventions efficacy to in-person care. Two meta-analyses (Hyler et al., 2005; Batastini et al., 2021) compared telepsychiatry to in-person care. They found no difference between the two modalities in terms of efficacy or patients’ satisfaction, therefore considering telepsychiatry a sensible option when in-person care is unsuitable. Nonetheless, more evidence-based research is warranted. Future research should monitor the long-term consequences of the pandemic on people with mental illness, along with defining specific intervention protocols to reach out all the patients.
